# Xenotransplantation: How close are we to clinical applications?

**DOI:** 10.1093/lifemedi/lnae037

**Published:** 2024-10-03

**Authors:** Hang Zhang, Yuan Chang, Jiangping Song

**Affiliations:** Beijing Key Laboratory of Preclinical Research and Evaluation for Cardiovascular Implant Materials, Animal Experimental Centre, National Centre for Cardiovascular Disease, Department of Cardiac Surgery, Fuwai Hospital, Chinese Academy of Medical Sciences and Peking Union Medical College, Beijing 100037, China; Beijing Key Laboratory of Preclinical Research and Evaluation for Cardiovascular Implant Materials, Animal Experimental Centre, National Centre for Cardiovascular Disease, Department of Cardiac Surgery, Fuwai Hospital, Chinese Academy of Medical Sciences and Peking Union Medical College, Beijing 100037, China; Beijing Key Laboratory of Preclinical Research and Evaluation for Cardiovascular Implant Materials, Animal Experimental Centre, National Centre for Cardiovascular Disease, Department of Cardiac Surgery, Fuwai Hospital, Chinese Academy of Medical Sciences and Peking Union Medical College, Beijing 100037, China

Xenotransplantation has recently made the leap from large animal models to preclinical and clinical research. In the USA, several preclinical studies have been conducted on the heart and kidneys of brain-dead patients. Recently, the University of Maryland achieved a milestone by preforming the first clinical transplantation of hearts from genetically modified pigs into two patients with heart failure [1]. In 2024, Massachusetts General Hospital achieved another landmark with the first successful xenotransplantation on a living patient with kidney failure, using the kidney from a gene-edited pig [2]. Concurrently in China, Dou Kefeng’s team successfully performed separate xenotransplantation of a pig liver and a pig kidney into two brain-dead patients.

Despite these promising preclinical and clinical achievements in xenotransplantation, transitioning to large-scale clinical applications presents novel challenges and introduces an additional layer of complexity. A fundamental question is the extent to which pig-derived organs can effectively regulate the complex physiology of humans over prolonged periods. Existing studies do not provide comprehensive evaluations and are not carried out over a sufficient period to assess the long-term impact.

For example, it is still questionable whether a porcine heart—even with the growth hormone receptor (GHR) knocked out—can withstand changes in human hemodynamics over an extended period. The doubts extend to kidney xenotransplantation. In most studies, assessments are often based on the kidney’s ability for urine production, while its ability to produce adrenal hormones is often not assessed. A recent study of kidney xenotransplantation performed on a brain-dead adult suggests that a porcine kidney could regulate mineral and bone metabolism, the renin-angiotensin-aldosterone system, and water balance, as well as process endogenous and exogenous solutes. However, the regulation of hormones in a living, functional organism is more complex, thereby casting doubt on its ability to regulate effectively in a living patient. Liver xenotransplantation presents even more, and significantly different challenges compared to hearts and kidneys. This is primarily due to severe dysregulation of coagulation and thrombocytopenia, which often lead to spontaneous hemorrhage. As such, preliminary clinical findings require further validation and replication in living patients, with a focus on evaluating more comprehensive functions and assessing long-term impacts, while also addressing organ-specific issues.

**Figure 1.**
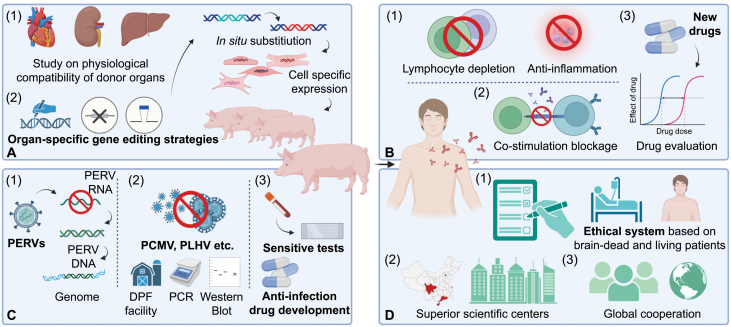
**Recent progress and potential development directions of xenotransplantation.** (A) In the acquisition of donor organs, it is essential to conduct a comprehensive analysis of physiological compatibility between the donor organs and the living recipients. A critical step in this process involves identifying the genes within various organs that require for modification. The implementation of *in situ* gene substitution could be highly beneficial. This approach could induce cell-specific expression of the target gene, thereby enhancing the compatibility and improving the success rate of the transplantation process. (B) In terms of immunosuppression, traditional immunosuppressive drugs continue to play a significant role. However, the use of the anti-CD40 antibody requires further evaluations due to potential unforeseen adverse complications. The development of new immunosuppressive regimens could be considered as potential alternative approaches. (C) Addressing pathogenic microorganisms, especially PERV and porcine cytomegalovirus (PCMV), is equally important. Early detection and blocking procedures for pathogenic microorganisms infection, via utilizing DPF facilities, antiviral drugs, polymerase chain reaction (PCR), and Western blot, are crucial to ensure the long-term survival of transplant recipients. (D) As xenotransplantation advances in China, it is critical to develop an ethical system that respects the unique culture and religious contexts. Furthermore, government policies are needed to strengthen domestic multicentre and international cooperation. These policies can help facilitate the transition of cutting-edge technologies and research findings into practical clinical applications. The graphic is created with Biorender.com.

Another set of challenges is how we can more efficiently and precisely obtain the source organs. Currently, the primary method for acquiring these organs involves multi-gene editing techniques on donor organs, such as CRISPR/Cas9. This technique improves donor-recipient compatibility, but generating donors with multiple gene edits is time-consuming—it requires several generations of gene-edited pigs to produce a homozygous individual. Further, replacing genes *in situ* is more beneficial for gene expression compared to integrating all sequences into a “safe harbor.” Using human-encoded sequences instead of porcine homologous genes can be considered for specific cell expression of target genes. This approach could facilitate cell-type-specific expression of edited genes, ensuring that only target cells (such as endothelial cells) express the edited genes, thus avoiding unnecessary gene expression in non-target cells. Still, an important question remains: is it essential to edit multiple genes, and if so, which specific genes should be targeted for modification? To ensure the stable functionality of inserted genes, it is common to simultaneously insert multiple genes with the same functions, which can lead to unpredicted and potentially harmful edits. For example, knocking out *CMAH* can lead to the production of new xenogeneic antigens, *GHR* knockout can increase liver phosphorylation metabolism [3], and the insertion of human *CD47* may cause systemic inflammation in recipients. Compared to multi-gene edited donors, α-Gal knockout (GTKO) donors have achieved considerable survival periods (61 days) in clinical kidney xenotransplantation experiments. Therefore, it is crucial to carefully weigh the pros and cons of gene editing in improving donor organ compatibility and its impact on donor organs when determining which genes need to be edited in different organs (Fig. 1A).

Embryonic chimeras present an innovative approach to mitigating the shortage of donor organs for xenotransplantation. Generating viable interspecies chimeras involves using embryo complementation with pluripotent stem cells (PSCs) to enable large mammals to support human tissues. This process allows the human tissues to occupy specific organogenesis-disabled niches in the host created through gene editing. A recent study has demonstrated the feasibility of this approach by successfully generating humanized mesonephroi in nephric-defective pigs through embryo complementation with PSCs. Although the gestation was terminated before the metanephros developed, the findings indicate that it might be possible to generate a functional human organ inside newborn pigs. Even with compatible source organs, immune rejection—particularly antibody-mediated immune rejection—remains a significant impediment to the survival of transplanted organs in recipients. The most effective and commonly adopted approach to suppressing the immune response involves depleting T and B lymphocytes in the recipient, followed by the administration of an anti-inflammatory treatment including antimetabolites and hormones after the transplantation. With the discovery of the critical role of the CD40–CD154 signaling pathway in antigen processing and B cell activation, immunosuppressive drugs targeting this pathway received attention in xenotransplantation. The first application of anti-CD154 monoclonal antibody significantly extended the survival period of xenografts, but due to its severe side effects—such as complications like platelet aggregation and thrombus formation—it was later replaced by anti-CD40 monoclonal antibodies, which then became the standard immunosuppressive therapy in xenotransplantation.

Despite its success in large animal models, the validity and effectiveness of blocking the CD40–CD154 costimulation in immunosuppressive therapy require further investigation in humans. To date, many clinical trials involving anti-CD40 monoclonal antibodies have been unsuccessful. These antibodies have also not yet received approval for clinical use—thus their usage is currently limited to small-scale clinical studies. Furthermore, maintaining controlled concentrations of anti-CD40 monoclonal antibodies in the blood during heart xenotransplantation presents a significant challenge [1]. High doses of these antibodies may increase the risk of infection, while lower doses may not effectively suppress immune rejection. Conversely, while similar doubts exist with anti-CD154 monoclonal antibodies, recent studies suggest anti-CD154 antibodies may enhance the transplant tolerance and durability, thereby preventing graft dysfunction and rejection [4]. In the second clinical trial of cardiac xenotransplantation conducted at the University of Maryland in the United States, anti-CD40 monoclonal antibodies were replaced with anti-CD154 monoclonal antibodies, but this change did not extend the survival time of the recipient. Future research should explore the potential benefits of replacing anti-CD40 antibody with more targeted and effective immunosuppressive regimens (Fig. 1B).

In addition to immunosuppressants, immune tolerance is another promising immunomodulatory regime. Inducing immune tolerance involves retraining the immune system of the recipients to recognize the donor organ as its own. At present, there are two primary approaches to inducing immune tolerance in recipients. One method is cotransplanting thymic tissue along with another organ. As a biological foundation of central tolerance, thymic tissue has the capability to induce tolerance to any other organ or tissue from the same donor. A recent clinical trial demonstrated that GTKO kidney xenografts from pigs, when transplanted combined with pig thymus, remained viable and functional in brain-dead human recipients without signs of hyperacute rejection. Another approach to inducing immune tolerance is “mixed chimerism.” This can be achieved by transferring donor hematopoietic cells to a recipient that has been immunosuppressed. This method has successfully promoted immune tolerance in humanized rodent models.

Xenotransplantation also presents various biosafety concerns that require thorough consideration. While some issues—such as the Porcine Endogenous Retrovirus (PERV)—have been largely mitigated through recent advancements in genetic editing techniques, which enable the suppression or complete deletion of PERV gene loci in the animals [5], the potential for infection from other pathogenic microorganisms originating from pigs continues to be a significant concern. This was illustrated in the first clinical trial of cardiac xenotransplantation, where the recipient developed Porcine Cytomegalovirus (CMV) viremia after the procedure [1]. While the potential infections can be mitigated through the development of more sensitive screening methodologies—including serological assays and PCR-based assays—it is crucial to systematically and comprehensively consider the production of CMV-free pigs and more effective antiviral treatment regimens for pig viruses. The establishment of designated pathogen‐free (DPF) facilities, coupled with the implementation of comprehensive microbial monitoring of donor animals, recipients, and their close contacts, will further enhance biosafety in xenotransplantation (Fig. 1C).

Despite the various challenges currently present in the field of xenotransplantation, recent advancements—particularly the extended survival rates observed in pig-to-non-human primates (NHPs) xenotransplantation and the progression of pre-clinical and clinical studies—have sparked a newfound optimism within this realm. These promising developments underscore the need for further large-scale preclinical and clinical studies to validate and replicate the initial findings. Nonetheless, the pursuit of such expansive studies in xenotransplantation is currently hindered by a limited sample size—due to a small number of brain-dead patients willing to contribute to research—and by substantial individual differences among these recipients. Therefore, it becomes imperative to establish a more rapid, reliable, and human-like donor and drug evaluation and screening platform to expediate the clinical transition process of xenotransplantation.

As xenotransplantation advances in China, it is crucial to build an ethical system that aligns with the nation’s ethical norms, reinforced by an efficient review and assessment procedure of ethic. At the policy level, it is essential for the government to give full play to the guiding role of national laboratories, the organizing role of national research institutes to facilitate the conversion of state-of-the-art technologies and research outcomes into practical clinical applications. Sustainable funding is needed to ensure both the continued implementation of research programs and continued support for those programs once they are implemented. This procedure should not only expediate the implementation of clinical studies but also ensure the protection of the rights and welfare of the participants (Fig. 1D).
